# The association between perceived discrimination and depression/anxiety disorders among Korean workers: analysis of the third Korean Working Conditions Survey

**DOI:** 10.1186/s40557-016-0121-4

**Published:** 2016-08-02

**Authors:** Sang Hyun Lee, Hee Sung Lee, Guang Hwi Kim, June-Hee Lee, Kyung-Jae Lee, Joo Ja Kim

**Affiliations:** Department of Occupational & Environmental Medicine, Soonchunhyang University Hospital, Seoul, Korea

**Keywords:** Discrimination, Depression, Anxiety disorder

## Abstract

**Background:**

Discrimination is a long-standing social problem, and interest in the health effects of discrimination has been increasing. Unfortunately, Korean workers experience various types and combinations of discrimination. This study aimed to examine the association between perceived discrimination and depression/anxiety disorders among Korean workers.

**Methods:**

Data from 33,530 paid workers were extracted from the third Korean Working Conditions Survey. The data included general characteristics, occupational characteristics, perceived discrimination, and depression/anxiety disorders. To examine the relationship between perceived discrimination and depression/anxiety disorders, multiple logistic regression analysis was used to evaluate depression/anxiety disorders as the dependent variable and perceived discrimination as the independent variable, after adjusting for relevant general and occupational characteristics.

**Results:**

After adjusting for the relevant general and occupational characteristics, we observed that male and female workers who had experienced perceived discrimination exhibited a significantly higher likelihood of having depression/anxiety disorders. The odds ratios among male and female workers were 3.25 (95 % confidence interval: 2.45–4.32) and 4.56 (95 % confidence interval: 3.45–6.03), respectively.

**Conclusions:**

Perceived discrimination was significantly related to depression or anxiety disorders among Korean workers. The risk of depression or anxiety was higher among female workers, compared to male workers.

## Background

Mental disorders among modern humans have received significant attention as a social problem, and, according to a recent survey, approximately 17 % of South Korean adults have experienced some form of a mental disorder. Among these disorders, the prevalences of major depressive disorder and anxiety disorder were especially high (6.7 and 8.7 %, respectively) [1]. Depression is the most representative mental disorder and the World Health Organization is predicting that depression will have the second highest global burden of disease by 2020, with ischemic heart disease having the greatest burden [2]. Furthermore, approximately 15 % of the general population have experienced anxiety disorder at least once during their lifetime [3]. If not properly treated, this disease can easily become chronic and can seriously affect a patient’s quality of life, which highlights the importance of prevention and management.

Koreans currently recognize that discrimination is a serious social problem. In 2004, a survey of 2000 Koreans found that 54.5 % of the respondents believed that discrimination was a serious problem in the Korean society, and that the most serious forms of discrimination were against disabled, under-educated, and foreign workers [4]. In addition, a 2003 survey of disabled people found that 73.3 % of the respondents indicated that they had been discriminated against based on their disability, and a case study by the National Human Rights Commission of the Republic of Korea reported that employment-related disability discrimination accounted for 14.5 % of all cases [5]. Furthermore, academic cliquism is a primary cause of various forms of discrimination that Koreans face in the workplace [6]. Moreover, foreign workers experience a greater level of discrimination in their pay, working hours, and work environment, compared to Korean workers [7]. Finally, discrimination based on birth origin, rank, and employment status are also being discussed as major social issues.

Discrimination refers to unreasonable exclusion or unfair treatment during an individual’s daily life or employment because of their inherent and/or social characteristics [4]. People who are discriminated against are socially excluded, which can lead to inequality, conflict between members of society, and negative effects on a person’s physical and mental health [8]. Thus, the health effects of perceived discrimination are considered an important topic in Korea and abroad [9]. In particular, a recent meta-analysis found that perceived discrimination was associated with mental health conditions, such as depression [9], and the effects of perceived discrimination on mental health are considered important issues in Korean studies [10]. In this context, the biological mechanism through which perceived discrimination affects mental health is known to involve activation of the hypothalamic-pituitary-adrenal axis and the sympathetic nervous system [11].

The workplace is a place where people spend a large part of their social life and form various interpersonal relationships with their superiors, coworkers, and other individuals. Thus, various combinations of discrimination based on sex, age, academic cliquism, employment status, and birth origin can occur [12], and can affect decisions regarding hiring, promotion, education, and normal work activities. As various studies have confirmed that discrimination can affect physical and mental health, identifying the current state of discrimination in the workplace is an important component of promoting health in the workplace. Previous Korean studies have found that discrimination based on age, disability, and sex resulted in unfair practices, and that discrimination was associated with self-reported disease and absenteeism [12]. Kim et al. have also reported that perceived discrimination was significantly associated with poor self-rated health when a person was being hired, receiving income, and after they had been laid off [6]. Moreover, Alvarez-Galvez et al. have reported that age-, disability-, and sex-based perceived discrimination was associated with poor health outcomes [13]. However, there are few studies regarding discrimination among workers, and even fewer studies have evaluated the subtypes of discrimination. Therefore, the present study aimed to identify the distributions of workplace discrimination, and to examine its association with depressive and anxiety disorders.

## Methods

### Study subjects

The present study used the data from the third Korean Working Conditions Survey (KWCS), which was conducted from June 1 to November 30, 2011 by the Korea Occupational Safety and Health Agency. This survey was initially conducted in 2006, and is a one-on-one household interview survey of working people who are ≥15 years old. The KWCS aims to provide information regarding the overall working conditions among Korean workers, to identify work-related diseases and accidents, and to establish safe and healthy working conditions for all workers. A total of 50,032 workers were surveyed in the third KWCS, although we excluded individuals with incomplete responses, military personnel, and individuals who worked in the fishery and forestry industries. After excluding these individuals, we included 33,530 paid workers who were ≥20 years old and worked in a workplace with ≥2 employees.

### Measurements

#### General characteristics

The general characteristics included sex, age (20–29 years, 30–39 years, 40–49 years, 50–59 years, or ≥60 years), educational status (middle school or less, high school, college, and university or above), smoking (current smoker, ex-smoker, never smoker), and alcohol drinking (yes or no).

#### Occupational characteristics

The occupational characteristics included employment status (regular worker, temporary worker, or day laborer), occupation type (management/professional, regular office work, service/sales, technical, or simple labor), monthly income (<1,500,000 Korean won [KRW], 1,500,000–1,990,000 KRW, 2,000,000–2,990,000 KRW, or ≥3,000,000 KRW), number of employees (<5, 5–49, 50–299, or ≥300), tenure (<2, 2–4, 5–9, or ≥10 years), working hours per week (≤40, 41–48, 49–60, >60 h), and shift work (yes or no).

#### Perceived discrimination

Perceived discrimination was evaluated using the question “Have you experienced any of the following work-related discrimination during the past 12 months?” The respondents were asked to answer “yes” or “no” to the following discrimination subtypes: age, academic cliquism, birth origin, sex, and employment status. Individuals who had experienced perceived discrimination were defined as anyone who answered “yes” regarding any of the five subtypes.

#### Depression/anxiety disorder

Depression/anxiety disorder was evaluated using the question “Have you had any of the following health issues during the past 12 months?” The respondents were asked to answer “yes” or “no” to the subtypes of depression or anxiety disorders. Individuals who had experienced depression or anxiety disorder were defined as anyone who responded “yes”.

### Data analysis

The chi-square test was used to identify difference in the distributions of the general and occupational characteristics based on perceived discrimination. Next, we analyzed the association between depression/anxiety disorders and the general and occupational characteristics. Depression/anxiety disorders were set as the dependent variable, and perceived discrimination was set as the independent variable. Multiple logistic regression analysis was performed after adjusting for the significant general and occupational characteristics from the univariate analyses, as well as characteristics that were associated with depression in previous studies. All analyses were stratified to identify sex-based differences. The level of statistical significance was set at α = 0.05, and all statistical analyses were performed using SPSS software (version 14.0; SPSS Inc., Chicago, IL).

## Results

### General and occupational characteristics of the study subjects

The 33,530 subjects included 20,020 (59.7 %) male subjects and 13,510 (40.3 %) female subjects. Among both male and female subjects, the largest age group was 30–39 years old and the smallest age group was ≥60 years old. The most common education level was a high school education, and the least common education level was middle school or below. Both male and female subjects were more likely to drink (vs. no drinking), although 53.8 % of the male subjects were current smokers, compared to only 5.9 % of the female subjects.

The largest proportions of male and female subjects were regular workers, although female subjects exhibited a higher frequency of being non-regular workers (vs. male subjects), which included temporary workers and day laborers. The highest percentage of male subjects worked in the technical field (30.0 %), whereas the highest percentage of female subjects worked in service/sales fields (29.9 %), which highlighted sex-specific differences in occupational types. Female subjects more frequently received a lower income (vs. male subjects), although both male and female subjects were most likely to work at a workplace with 5–49 workers. Both male and female subjects were most likely to have worked for <2 years, although male subjects exhibited a higher frequency of having a tenure of ≥10 years (23.9 vs. 8.0 % among female subjects). Both male and female subjects were most likely to work <40 h/week, although female subjects exhibited a higher frequency of working <40 h/week (45.4 vs. 36.4 % among male subjects). Male subjects were more likely to have shift work (Table 1).

**Table 1:** General and occupational characteristics of the individuals according to perceived discrimination

	Male (*N* = 22,020)			Female (*N* = 13,510)		
		Perceived discrimination			Perceived discrimination	
	*N* (%)	No (%)	Yes (%)	*N* (%)	No (%)	Yes (%)
General characteristics						
Age (years)						
20–29	2331 (11.6)	2050 (87.9)	281 (12.1)*	2767 (20.5)	2410 (87.1)	357 (12.9)
30–39	6456 (32.2)	5749 (89.0)	707 (11.0)	4123 (30.5)	3556 (86.2)	567 (13.8)
40–49	5963 (29.8)	5335 (89.5)	628 (10.5)	4006 (29.7)	3472 (86.7)	534 (13.3)
50–59	3718 (18.6)	3258 (87.6)	460 (12.4)	1806 (13.4)	1572 (87.1)	234 (12.9)
≥ 60	1552 (7.8)	1345 (86.7)	207 (13.3)	808 (6.0)	697 (86.3)	111 (13.7)
Education						
Middle school or below	1688 (8.4)	1432 (84.8)	256 (15.2)*	1520 (11.3)	1311 (86.2)	210 (13.8)*
High school	7214 (36.0)	6373 (88.3)	841 (11.7)	4941 (36.6)	4336 (87.8)	605 (12.2)
College^a^	3234 (16.2)	2812 (87.0)	422 (13.0)	2873 (21.3)	2473 (86.1)	400 (13.9)
University or above	7884 (39.4)	7120 (90.3)	764 (9.7)	4176 (30.9)	3587 (85.9)	588 (14.1)
Alcohol drinking						
No	2751 (13.7)	2414 (87.7)	337 (12.3)	4605 (34.1)	4066 (88.3)	539 (11.7)*
Yes	17,269 (86.3)	15,323 (88.7)	1946 (11.3)	8905 (65.9)	7642 (85.8)	1263 (14.2)
Smoking						
Nonsmoker	5875 (29.3)	5196 (88.4)	679 (11.6)	12,304 (91.1)	10,755 (87.4)	1549 (12.6)*
Ex-smoker	3380 (16.9)	3009 (89.0)	371 (11.0)	407 (3.0)	343 (84.3)	64 (15.7)
Current smoker	10,765 (53.8)	9532 (88.5)	1233 (11.5)	799 (5.9)	609 (76.3)	190 (23.8)
Occupational characteristics						
Employment status						
Regular worker	16,983 (84.8)	15,188 (89.4)	1795 (10.6)*	10,454 (77.4)	9095 (87.0)	1359 (13.0)
Temporary worker	1933 (9.7)	1691 (87.5)	242 (12.5)	2421 (17.9)	2062 (85.2)	359 (14.8)
Day laborer	1104 (5.5)	858 (77.7)	246 (22.3)	635 (4.7)	551 (86.8)	84 (13.2)
Occupation type						
Management/professional	3835 (19.2)	3429 (89.4)	406 (10.6)*	3424 (25.3)	2941 (85.9)	483 (14.1)*
Office work	5104 (25.5)	4580 (89.7)	524 (10.3)	3252 (24.1)	2790 (85.8)	462 (14.2)
Service/sales	2444 (12.2)	2202 (90.1)	242 (9.9)	4040 (29.9)	3569 (88.3)	471 (11.7)
Technical	6009 (30.0)	5343 (88.9)	666 (11.1)	948 (7.0)	824 (86.9)	124 (13.1)
Simple labor	2628 (13.1)	2184 (83.1)	444 (16.9)	1846 (13.7)	1583 (85.8)	263 (14.2)
Income (/10,000/month)						
< 150	2809 (14.0)	2401 (85.5)	408 (14.5)*	6064 (44.9)	5358 (88.4)	706 (11.6)*
150–199	3626 (18.1)	3110 (85.8)	516 (14.2)	3435 (25.4)	2977 (86.7)	458 (13.3)
200–299	7078 (35.4)	6311 (89.2)	767 (10.8)	2634 (19.5)	2231 (84.7)	403 (15.3)
≥ 300	6508 (32.5)	5916 (90.9)	592 (9.1)	1377 (10.2)	1142 (82.9)	235 (17.1)
Number of employees						
< 5	3342 (16.7)	3023 (90.5)	319 (9.5)*	3923 (29.0)	3560 (90.7)	363 (9.3)*
5–49	10,557 (52.7)	9283 (87.9)	1274 (12.1)	7045 (52.1)	6044 (85.8)	1001 (14.2)
50–299	4067 (20.3)	3579 (88.0)	488 (12.0)	1984 (14.7)	1653 (83.3)	331 (16.7)
≥ 300	2054 (10.3)	1852 (90.2)	202 (9.8)	558 (4.1)	450 (80.6)	108 (19.4)
Tenure (years)						
< 2	6007 (30.0)	5256 (87.5)	751 (12.5)*	6405 (47.4)	5579 (87.1)	826 (12.9)
2–4	4840 (24.2)	4257 (88.0)	583 (12.0)	4037 (29.9)	3474 (86.1)	563 (13.9)
5–9	4391 (21.9)	3928 (89.5)	463 (10.5)	1991 (14.7)	1719 (86.3)	272 (13.7)
≥ 10	4782 (23.9)	4296 (89.8)	486 (10.2)	1077 (8.0)	935 (86.8)	142 (13.2)
Working hours (/week)						
≤ 40	7285 (36.4)	6616 (90.8)	669 (9.2)*	6139 (45.4)	5355 (87.2)	784 (12.8)*
41–48	4403 (22.0)	3921 (89.1)	482 (10.9)	3111 (23.0)	2724 (87.6)	387 (12.4)
49–60	6248 (31.2)	5409 (86.6)	839 (13.4)	3327 (24.6)	2843 (85.4)	484 (14.6)
> 60	2084 (10.4)	1792 (86.0)	292 (14.0)	933 (6.9)	786 (84.3)	147 (15.7)
Shift work						
No	17,673 (88.3)	15,697 (88.8)	1976 (11.2)*	12,688 (93.9)	11,047 (87.1)	1641 (12.9)*
Yes	2347 (11.7)	2040 (86.9)	307 (13.1)	822 (6.1)	660 (80.3)	162 (19.7)

*p < 0.05 using the chi-square test

^a^a 2–3-year college degree

### Perceived discrimination according to general and occupational characteristics

Among the various age groups, male subjects who were ≥60 years old were most likely to have experienced perceived discrimination, although there was no age-specific difference among female subjects. Among male subjects, the rate of perceived discrimination was highest at the education level of middle school or below; among female subjects, the rate of perceived discrimination was highest at the education level of university or higher. Among female subjects, the rates of perceived discrimination were highest in the groups that drink alcohol (14.2 %) and were current smokers (23.8 %), although no significant differences were observed for the male subjects.

Among male subjects, the rate of perceived discrimination was highest among day laborers (22.3 %), although there was no significant difference among female subjects according to employment status. Male subjects exhibited the highest rate of perceived discrimination among simple laborers, although female subjects exhibited high rates of perceived discrimination in the management/professional, office work, and simple labor categories. Among the male subjects, the group that earned <1.5 million KRW had the highest rate of perceived discrimination (14.5 %), although female subjects who earned ≥3 million KRW had the highest rate of perceived discrimination (17.1 %). The rates of perceived discrimination were highest among men who worked in workplaces with 5–29 employees (12.1 %) and among female subjects who worked in workplaces with ≥300 employees (19.4 %). Among male subjects, the rate of perceived discrimination was highest in the group with a tenure of <2 years (12.5 %), although no tenure-specific difference was observed among the female subjects. Both male and female subjects exhibited the highest rate of perceived discrimination when they worked >60 h/week, and shift workers exhibited a higher rate of perceived discrimination, compared to non-shift workers (Table 1).

### Differences in general and occupational characteristics according to depression/anxiety disorders

Depression or anxiety disorders were self-reported by 1.2 % of the male subjects and 1.7 % of the female subjects. Among the female subjects, the highest rates of depression/anxiety disorders were observed among workers who were ≥60 years old (3.0 %), educated to a high school level or below (2.0 %), and current smokers (2.6 %). No significantly differences in the rates of depression/anxiety disorders were observed among the male subjects. Furthermore, both male and female subjects exhibited no alcohol consumption-specific differences.

The incidences of depression/anxiety disorder were highest among female subjects who held technical jobs (3.6 %) and who worked in a workplace with 50–299 employees (3.0 %). However, there was no significant job-specific difference among the male subjects. The highest incidences of depression/anxiety disorder were observed among male subjects with 2–4 years of tenure (1.6 %) and among female subjects with ≥10 years of tenure (3.1 %). The highest incidences of depression/anxiety disorder were observed among male subjects who worked 49–60 h/week (1.6 %) and among female subjects who worked 41–48 h/week (2.2 %). Both male and female subjects exhibited no significant differences in depression/anxiety disorders based on employment status, income, and shift work (Table 2).

**Table 2:** Distribution of depression/anxiety disorders according to general and occupational characteristics

	Depression/anxiety disorders					
	Male (*N* = 22,020)			Female (*N* = 13,510)		
	No (%)	Yes (%)	*P*-value^a^	No (%)	Yes (%)	*P*-value^a^
Total	19,778 (98.8 %)	242 (1.2 %)	0.001*	13,286 (98.3 %)	224 (1.7 %)	0.001*
General characteristics						
Age (years)			0.072			<0.001*
20–29	2303 (98.8)	29 (1.2)		2739 (99.0)	28 (1.0)	
30–39	6369 (98.7)	87 (1.3)		4066 (98.6)	57 (1.4)	
40–49	5904 (99.0)	58 (1.0)		3919 (97.8)	87 (2.2)	
50–59	3664 (98.5)	55 (1.5)		1777 (98.4)	29 (1.6)	
≥ 60	1539 (99.2)	12 (0.8)		784 (97.0)	24 (3.0)	
Education			0.360			<0.001*
Middle school or below	1672 (99.0)	17 (1.0)		1490 (98.0)	30 (2.0)	
High school	7113 (98.6)	100 (1.4)		4844 (98.0)	97 (2.0)	
College^b^	3199 (98.9)	35 (1.1)		2853 (99.3)	21 (0.7)	
University or above	7794 (98.9)	90 (1.1)		4099 (98.2)	76 (1.8)	
Alcohol drinking			0.587			0.345
No	2716 (98.7)	36 (1.3)		4522 (98.2)	83 (1.8)	
Yes	17,063 (98.8)	205 (1.2)		8764 (98.4)	141 (1.6)	
Smoking			0.465			0.033*
Nonsmoker	5801 (98.7)	75 (1.3)		12,111 (98.4)	193 (1.6)	
Ex-smoker	3333 (98.6)	46 (1.4)		397 (97.5)	10 (2.5)	
Current smoker	10,644 (98.9)	121 (1.1)		778 (97.4)	21 (2.6)	
Occupational characteristics						
Employment status			0.467			0.257
Regular worker	16,774 (98.8)	209 (1.2)		10,290 (98.4)	165 (1.6)	
Temporary worker	1915 (99.1)	18 (0.9)		2376 (98.2)	44 (1.8)	
Day laborer	1089 (98.6)	15 (1.4)		620 (97.6)	15 (2.4)	
Occupation type			0.055			<0.001*
Management/professional	3773 (98.4)	62 (1.6)		3383 (98.8)	40 (1.2)	
Office work	5047 (98.9)	57 (1.1)		3207 (98.6)	45 (1.4)	
Service/sales	2421 (99.1)	23 (0.9)		3973 (98.3)	68 (1.7)	
Technical	5933 (98.7)	76 (1.3)		914 (96.4)	34 (3.6)	
Simple labor	2604 (99.1)	24 (0.9)		1808 (97.9)	38 (2.1)	
Income (/10,000/month)			0.086			0.426
< 150	2762 (98.3)	47 (1.7)		5961 (98.3)	103 (1.7)	
150–199	3581 (98.8)	45 (1.2)		3386 (98.6)	48 (1.4)	
200–299	7003 (98.9)	75 (1.1)		2588 (98.3)	46 (1.7)	
≥ 300	6432 (98.8)	75 (1.2)		1350 (98.0)	28 (2.0)	
Number of employees			0.081			<0.001*
< 5	3305 (98.9)	36 (1.1)		3874 (98.8)	48 (1.2)	
5–49	10,439 (98.9)	118 (1.1)		6947 (98.6)	99 (1.4)	
50–299	4003 (98.4)	65 (1.6)		1925 (97.0)	59 (3.0)	
≥ 300	2032 (98.9)	22 (1.1)		540 (96.8)	18 (3.2)	
Tenure (years)			0.019*			0.002*
< 2	5941 (98.9)	66 (1.1)		6309 (98.5)	97 (1.5)	
2–4	4763 (98.4)	77 (1.6)		3976 (98.5)	61 (1.5)	
5–9	4336 (98.7)	55 (1.3)		1957 (98.3)	33 (1.7)	
≥ 10	4738 (99.1)	44 (0.9)		1044 (96.9)	33 (3.1)	
Working hours (/week)			0.002*			0.034*
≤ 40	7221 (99.1)	64 (0.9)		6055 (98.6)	84 (1.4)	
41–48	4354 (98.9)	49 (1.1)		3044 (97.8)	67 (2.2)	
49–60	6149 (98.4)	99 (1.6)		3273 (98.4)	54 (1.6)	
> 60	2055 (98.6)	29 (1.4)		914 (98.0)	19 (2.0)	
Shift work			0.703			0.125
No	17,467 (98.8)	36 (1.5)		12,478 (98.4)	209 (1.6)	
Yes	2311 (98.5)	206 (1.2)		808 (98.2)	15 (1.8)	

*p < 0.05

^a^Calculated using the chi-square test

^b^a 2–3-year college degree

### Rate of perceived discrimination based on sex

Among all respondents, 11.4 % of the male subjects and 13.3 % of the female subjects indicated that they had experienced at least one form of discrimination (age, academic cliquism, birth origin, sex, and employment status). Female subjects exhibited higher incidences of perceived discrimination for all types of discrimination, compared to male subjects, and this difference was especially pronounced for sex-based discrimination (female subjects: 3.3 %, male subjects: 1.8 %). Both male and female subjects exhibited the highest rates of discrimination for academic cliquism, and the lowest rates for birth origin. Subjects who experienced discrimination exhibited a significantly higher rate of depression/anxiety disorders, regardless of the type of discrimination (Table 3).

**Table 3:** Perceived discrimination according to depression/anxiety disorders

	Male (*N* = 22,020)			Female (*N* = 13,510)		
		Depression/anxiety disorders			Depression/anxiety disorders	
Perceived discrimination	*N* (%)	No (%)	Yes (%)	*N* (%)	No (%)	Yes (%)
Total						
Yes	2284 (11.4)	2212 (96.8)	72 (3.2)*	1802 (13.3)	1711 (95.0)	91 (5.0)*
No	17,736 (88.6)	17,566 (99.0 %)	170 (1.0)	11,708 (86.7)	11,575 (98.9)	133 (1.1)
Age						
Yes	835 (4.2)	816 (97.7)	19 (2.3)*	635 (4.7)	589 (92.8)	46 (7.2)*
No	19,185 (95.8)	18,962 (98.8)	223 (1.2)	12,875 (95.3)	12,697 (98.6)	178 (1.4)
Academic cliquism						
Yes	1142 (5.7)	1110 (97.2)	32 (2.8)*	830 (6.1)	779 (93.9)	51 (6.1)*
No	18,878 (94.3)	18,668 (98.9)	210 (1.1)	12,680 (93.9)	12,506 (98.6)	174 (1.4)
Birth origin						
Yes	435 (2.2)	422 (97.0)	13 (3.0)*	306 (2.3)	286 (93.5)	20 (6.5)*
No	19,585 (97.8)	19,356 (98.8)	229 (1.2)	13,204 (97.7)	13,000 (98.5)	204 (1.5)
Sex						
Yes	369 (1.8)	353 (95.7)	16 (4.3)*	446 (3.3)	404 (90.6)	42 (9.4)*
No	19,651 (98.2)	19,426 (98.9)	225 (1.1)	13,064 (96.7)	12,882 (98.6)	182 (1.4)
Employment status						
Yes	834 (4.2)	799 (95.8)	35 (4.2)*	654 (4.8)	600 (91.7)	54 (8.3)*
No	19,186 (95.8)	18,979 (98.9)	207 (1.1)	12,856 (95.2)	12,686 (98.7)	170 (1.3)

*p < 0.05 using the chi-square test

### Association between perceived discrimination and depression/anxiety disorder

Age, education, smoking, occupation type, number of employees, tenure, and working hours exhibited significant relationships with the incidence of depression/anxiety disorders. Thus, these factors were incorporated in the multiple logistic regression analysis, as well as shift work and alcohol consumption (factors that were associated with depression in previous studies).

The odds ratios (OR) for depression/anxiety disorder among male and female subjects with perceived discrimination were 3.25 (95 % confidence interval [CI]: 2.45–4.32) and 4.56 (95 % CI: 3.45–6.03); female subjects exhibited a higher OR. Significant results were found for all discrimination types among male subjects: age-based discrimination (OR: 1.98, 95 % CI: 1.22–3.20), academic cliquism (OR: 2.42, 95 % CI: 1.65–3.55), birth origin-based discrimination (OR: 2.63, 95 % CI: 1.49–4.64), sex-based discrimination (OR: 3.78, 95 % CI: 2.25–6.35), and employment status discrimination (OR: 4.03, 95 % CI: 2.78–5.84). Significant results were also found for all discrimination types among female subjects: age-based discrimination (OR: 4.89, 95 % CI: 3.45–6.94), academic cliquism (OR: 4.76, 95 % CI: 3.40–6.67), birth origin-based discrimination (OR: 3.99, 95 % CI: 2.43–6.55), sex-based discrimination (OR: 7.75, 95 % CI: 5.36–11.20), and employment status discrimination (OR: 6.94, 95 % CI: 4.98–9.67). Female subjects exhibited especially high ORs for sex-based and employment status discrimination (Table 4).

**Table 4:** Odds ratios for depression/anxiety disorders according to perceived discrimination

Perceived discrimination	Male		Female	
	OR	(95 % CI)	OR	(95 % CI)
Total				
No	1.00		1.00	
Yes	3.25	2.45–4.32	4.56	3.45–6.03
Subtype				
Age				
No	1.00		1.00	
Yes	1.98	1.22–3.20	4.89	3.45–6.94
Academic cliquism				
No	1.00		1.00	
Yes	2.42	1.65–3.55	4.76	3.40–6.67
Birth origin				
No	1.00		1.00	
Yes	2.63	1.49–4.64	3.99	2.43–6.55
Sex				
No	1.00		1.00	
Yes	3.78	2.25–6.35	7.75	5.36–11.20
Employment status				
No	1.00		1.00	
Yes	4.03	2.78–5.84	6.94	4.98–9.67

Calculated using multiple logistic regression analysis (adjusted for age, education, alcohol drinking, smoking, occupation type, number of employees, tenure, work hours, and shift work)

*OR* odds ratio, *CI* confidence interval

## Discussion

In August 2015, 67.5 % of all Korean paid workers were regular workers and 32.5 % were non-regular workers. However, female workers were more likely to be non-regular workers (40.2 %), compared to male workers (26.5 %) [14]. Although women are becoming more likely to participate in economic activities, men are still recognized as the primary breadwinner. Furthermore, the patriarchal social perception suggests that women are responsible for housework and child rearing, which may have resulted in female workers seeking non-regular employment and/or hourly work. Moreover, the industrial structure has recently changed after the economic crisis to reduce the focus on manufacturing and to increase the focus on the service sector and a flexible labor market strategy, which might further drive female workers to have non-regular employment [15]. Therefore, these changes have driven female workers into a vulnerable tier within the labor market, which exposes them to multiple types of discrimination (e.g., age-, sex-, and employment status-based discrimination regarding wages, benefits, and working conditions) and greater discrimination, compared to their male or regular worker counterparts [16].

The present study investigated the distribution of discrimination among Korean workers, examined the general and occupational characteristics that are associated with depression/anxiety disorders, and evaluated the association between perceived discrimination and depression/anxiety disorders. Multiple logistic regression analyses revealed that depression/anxiety disorders were associated with all types of perceived discrimination, and that female subjects exhibited especially high ORs for these disorders. In this context, discrimination based on a person’s social identity increases their awareness of this mistreatment, and aggravated awareness of mistreatment can ultimately lead to chronic stress [17–20]. Therefore, perceived discrimination can act as a stressor that induces neurological disorders, such as depression [11].

In the present study, female subjects exhibited a higher rate of perceived discrimination (vs. male subjects), and even stronger associations with depression/anxiety disorders that were observed in the multiple logistic regression analysis. Therefore, female workers may experience depression/anxiety disorders because they experience discrimination. However, even female subjects who were university graduates, held a professional occupation, or had relatively high incomes (>3,000,000 KRW/month) exhibited higher rates of perceived discrimination, compared to their male counterparts. These findings suggest that even female workers with a relatively high socioeconomic status can be exposed to unfair treatment at work, such as sex-based discrimination.

In the present study, female subjects exhibited higher ORs for depression/anxiety disorder and age-based discrimination (e.g., having an age limit for hiring). According to the industry status survey by the Korean Labor Institute, 50 % of businesses had an age limit for new hires and 58.6 % of businesses avoided hiring elderly applicants. Furthermore, age was used as a criterion for honorary retirement or layoff in companies that were undergoing employment adjustments [21]. In this context, perceived age-based discrimination can include discriminatory expressions regarding age, apathy, lack of recognition, and the use of inappropriate words [22]. Thus, hiring practices that are influenced by age-based discrimination can negatively affect the applicant’s health and economic status.

Female subjects also exhibited a stronger association between perceived academic cliquism and depression/anxiety disorders. If education level is viewed according to matriculation of institutional education, academic cliquism can be viewed as a concept that makes the alma mater a hotbed for signalism, hierarchy, and factionalism [23]. This concept is supported by education-based discrimination and academic cliquism in the labor market, as a survey by the Korean Research Institute for Vocational Education & Training found that 64 % of human resources managers reported that academic cliquism influenced their hiring decisions [24]. Furthermore, a Ministry of Employment and Labor survey regarding education-based pay structure found that, for every 100 KRW earned by high school graduates, university graduates earned 150.9 KRW and vocational college graduates earned 103.4 KRW. Moreover, education-based discrimination has been reported as the primary source of discrimination among Korean male and female workers [6], and this association is further supported by our findings that education-based discrimination exhibited the highest rate among the 5 discrimination subtypes in the present study.

In the present study, the rate of perceived birth origin-based discrimination was higher among female subjects, although it had the lowest rate among the 5 discrimination subtypes. In this context, male workers usually experience discrimination based on birth origin during promotion [6]. Furthermore, a previous study evaluated differences in pay level based on birth origin, and found that the problem was not emotional in nature (e.g., an aversion to a specific birth origin), but was related to relative gaps in management/executive recruitment [25]. Moreover, birth origin-based discrimination was significantly associated with depression/anxiety disorder in the present study, although the absolute rate was relatively low.

Sex-based discrimination was most common among female subjects (vs. male subjects), although the rate of sex-based discrimination was relatively low (only birth origin-based discrimination was less frequent). This finding conflicts with previous findings that sex-based discrimination is the most common form that is experienced by female workers [6]. However, it appears that most female workers were employed in workplaces that also employed large numbers of other female workers, as the OR for sex-based discrimination’s association with depression/anxiety disorders among female workers was very high (OR: 7.75, 95 % CI: 5.36–11.20). Moreover, female workers are in a vulnerable labor group that may be exposed to multiple forms of discrimination (e.g., sexual molestation or sexual harassment).

Discrimination based on employment status was the second most common type of discrimination for both male and female subjects, although female subjects exhibited a higher rate. For example, the OR values for depression/anxiety disorders were 4.03 (95 % CI: 2.78–5.84) among male subjects and 6.94 (95 % CI: 4.98–9.67) among female subjects. Furthermore, a previous study found that regular workers were paid 15,289 KRW, whereas non-regular workers were paid 10,279 KRW, which corresponds to a 32.8 % pay rate reduction. However, female workers exhibited only a 19.8 % reduction, which indicates that female workers may experience a smaller pay discrepancy, compared to their male counterparts [16]. Nevertheless, the discriminatory treatment that non-regular workers actually experience is also considered present in their employment conditions and benefits, and is considered more prominent among female workers.

There are several limitations in this study. First, we used a cross-sectional design that cannot provide information regarding the causality of the relationship between perceived discrimination and depression/anxiety disorders. However, previous cross-sectional studies have also identified associations between perceived discrimination and health [6, 13, 26], and the findings of the present study support this relationship. Second, the survey question regarding perceived discrimination was simple and subjective, and did not require structured responses or provide a clear definition for each type of discrimination. Third, the question regarding depression/anxiety disorders was ambiguous, and it is unclear whether the responses were based on self-diagnosing or a clinical diagnosis. Nevertheless, a previous study used KWCS data and found associations between workplace injustice, such as discrimination, and an increased risk of self-reported disease [12].

Despite these limitations, the present study also has several strengths. First, we performed analyses of various types of discrimination according to sex, and confirmed that each discrimination type was associated with depression/anxiety disorders. Moreover, we found that female workers had a higher risk of depression/anxiety disorders, compared to male workers, and that various other groups had an increased risk of depression/anxiety disorders. However, future studies are needed to evaluate the health effects of various types of mistreatment, including discrimination, that may be experienced by each working population.

## Conclusions

This study revealed that perceived discrimination was related to depression/anxiety disorders among Korean paid workers, and that female workers had a higher risk of these disorders, compared to male workers. These findings may provide basic data for developing interventions to prevent discrimination in the workplace, although further research is needed to evaluate the various types of perceived discrimination and mental health conditions.
